# Latent profile analysis of nurses’ knowledge, attitude and practice regarding excessive oxygen therapy and influencing factors

**DOI:** 10.3389/fmed.2026.1798087

**Published:** 2026-06-05

**Authors:** Lingjun Luo, Linfei Li, Jingwen Zhang, Na Li, Zhongxian Yang, Chunchun Liu, Xianjuan Gou

**Affiliations:** Department of Emergency, Affiliated Hospital of Zunyi Medical University, Zunyi, Guizhou, China

**Keywords:** cross-sectional survey, excessive oxygen therapy, knowledge-attitudes-practices, latent profile analysis, nurses

## Abstract

**Objective:**

This study aims to assess the latent profile characteristics of Chinese nurses’ knowledge, attitudes, and practices regarding excessive oxygen therapy and explore the influencing factors of different profile types.

**Methods:**

From November to December 2025, convenience sampling was used to select nurses from secondary and tertiary general hospitals in Zunyi City, Guizhou Province, as the study participants. A self-designed demographic characteristics questionnaire and the Knowledge, Attitudes and Practice Scale of Healthcare Professionals on Excessive Oxygen Therapy (KAP-HPEOT) were used as survey tools. Latent profile analysis (LPA) was performed on the nurses’ KAP regarding excessive oxygen therapy. Univariate analysis of factors influencing different profiles was performed using the chi-square test and ANOVA, and multivariate analysis was conducted using logistic regression.

**Results:**

A total of 743 questionnaires were collected, with 687 valid responses, yielding an effective response rate of 92.46%. Latent profile analysis revealed that nurses’ knowledge, attitudes, and practices regarding excessive oxygen therapy could be categorized into three profile types: “Low Knowledge–Moderately Attitude–Moderate Practice” (19.65%), “Moderate Knowledge–Moderately Attitude–Moderate Practice” (20.23%), and “High Knowledge–Positive Attitude–High Practice” (60.12%). Logistic regression analysis indicated that age, work experience, professional title, education level, hospital level, participation in oxygen therapy training, and department were significant influencing factors of nurses’ KAP regarding excessive oxygen therapy (*P* < 0.005).

**Conclusion:**

This study confirms that nurses’ KAP regarding excessive oxygen therapy can be categorized into three profiles, and demographic-related characteristics significantly influence their KAP. The findings provide an important theoretical basis for nursing managers and educators to develop relevant training programs.

## 1 Introduction

Oxygen therapy is a medical treatment primarily aimed at patients experiencing hypoxia, which involves administering oxygen at a concentration higher than that in the atmosphere ( > 21%) (1). Excessive oxygen therapy refers to the provision of oxygen exceeding the physiological demand of patients, which can lead to hyperoxia (2), cause damage to vital organs such as the heart, brain and lungs, and even increase the mortality of patients (3). Therefore, standardizing clinical oxygen therapy practices and avoiding the risks of excessive oxygen therapy have become crucial steps in ensuring patient safety.

As frontline healthcare providers who directly administer oxygen therapy and monitor patients’ conditions, nurses’ knowledge, attitudes, and practices (KAP) regarding excessive oxygen therapies are critical to ensuring its safety and effectiveness. The KAP theory is commonly used to assess healthcare-related knowledge, attitudes, practices, and intervention acceptance (4). Based on the premise that knowledge informs attitudes and further drives practice (5). While previous research has explored oxygen therapy about KAP in healthcare providers, studies have shown variations in nurses’ KAP toward oxygen therapy. For instance, according to studies conducted in Ethiopia, the proportion of nurses with good knowledge ranged from 52 to 61.5% (6–8). In contrast, the proportions were extremely low in Yemen and Egypt, at 3 and 6% respectively (9, 10). Arasi et al. reported a much higher rate (87.0%) (11). Regarding practice, some studies showed that the rate of good practice in oxygen therapy among nurses ranged from 34.9 to 67.0% (6, 7, 12), while another study indicated that 97% of nurses demonstrated very proactive or positive practice behaviors (13).

Differences in nurses’ KAP regarding excessive oxygen therapy may be influenced by various factors. Individual factors such as educational background, work experience, and participation in professional training may lead to variations in knowledge levels and practical skills (14, 15). Organizational factors, including the accessibility of clinical guidelines, supervision and management systems, and the availability of monitoring equipment, also play a significant role in nurses’ practices (16). Additionally, departmental differences may contribute to variations in nurses’ KAP regarding excessive oxygen therapy as well. For example, nurses in intensive care units (ICUs) and emergency departments, who care for more critically ill patients and use oxygen therapy more frequently, may exhibit different cognitive and practical patterns compared to nurses in general wards (17, 18)

Currently, most relevant studies on nurses’ KAP toward excessive oxygen therapy regard nurses as an integrated group to investigate the KAP level and related influencing factors, while lacking exploration of the potential latent classifications of nurses’ KAP of excessive oxygen therapy.

Latent profile analysis (LPA) can provide a more scientific approach to classifying nurses’ KAP regarding excessive oxygen therapy. Its core advantage lies in fully accounting for inter-individual differences while adhering to the individual-centered research. LPA categorizes individuals according to their response patterns to external measurement items, which helps identify differences in participants’ responses across various items. This method also allows for the estimation of the proportion of individuals within each profile and provides a basis for further exploring the characteristics of distinct subgroups (19).

Therefore, this study aims to use latent profile analysis to explore the potential profiles of nurses’ KAP regarding excessive oxygen therapy and analyze the influencing factors of different profiles and to provide a theoretical basis for improving nurses’ awareness of excessive oxygen therapy, standardizing clinical oxygen therapy practices, reducing the occurrence of excessive oxygen therapy, and ultimately enhancing patient care quality and safety.

## 2 Materials and methods

### 2.1 Participants

This study is a cross-sectional study. From November to December 2025, convenience sampling was used to select nurses from secondary and tertiary general hospitals in Zunyi City as the study participants. The inclusion criteria were as follows: (1) holding a nurse practice certificate and having ≥ 1 year of clinical nursing experience; (2) voluntary participation in the study. The exclusion criteria were: nurses who were interns, trainees, or in standardized training programs. Additionally, nurses who were not on duty during the survey period due to reasons such as external training, illness, leave, or further education were excluded.

According to Kendall’s sample size estimation principle, the sample size should be 5–10 times the number of scale items. The required sample size for this study was *N* = 10 × 40 = 400. Considering a 10–20% attrition rate, the planned sample size was *N* = 400 ÷ (100–20%) = 500. As suggested in previous research, the minimum sample size for an LPA study is 300–500 (20). A total of 743 questionnaires were collected, with 687 valid responses, resulting in an effective response rate of 92.46%.

### 2.2 Instruments

#### 2.2.1 General information questionnaire

Based on a literature review and expert recommendations, a self-designed questionnaire was used, including eight items such as gender, age, professional title, education level, current department, work experience, hospital level, whether they have participated in oxygen therapy training.

#### 2.2.2 Knowledge, attitudes and practice of healthcare professionals on excessive oxygen therapy scale (KAP-HPEOT)

The KAP-HPEOT was developed by Ke et al. (21). It consists of three parts: knowledge, attitudes, and practice, with a total of 32 items. The knowledge dimension includes 15 items, items 1–9, 12, and 13 were single-choice questions, with 1 point awarded for a correct answer and 0 points for an incorrect or uncertain answer. Items 10, 11, 14, and 15 were multiple-choice questions, with 0 points for an incomplete or excessive selection and 1 point awarded for a fully correct answer. The total score was 15 points, with a higher score indicating a better mastery of knowledge. The attitude dimension comprised 6 items, rated on a 5-point Likert scale from 1 to 5 (1 = Strongly disagree, 5 = Strongly agree), with a total score ranging from 6 to 30; a higher score indicated a more positive attitude. The behavior dimension included 11 items, also assessed on a 5-point Likert scale from 1 to 5 (1 = Never, 5 = Always), with a total score of 11–55; a higher score reflected a higher frequency of adopting positive behaviors. The overall Cronbach’s α of the KAP-HPEOT was 0.889 in our study, demonstrating good reliability and validity.

### 2.3 Data collection

An electronic questionnaire was developed via the Wenjuanxing Platform for this study and was sent to head nurses in various hospitals through WeChat, with clear explanations of the research purpose, target respondents and questionnaire completion instructions provided. After obtaining participant’s informed consent, the electronic questionnaire was distributed for voluntary completion. The questionnaire was administered anonymously. The participants were required to complete all questions before submission, with each person allowed to submit only once, ensuring the completeness and validity of the questionnaire. A total of 743 questionnaires were collected, with 687 valid responses, yielding an effective response rate of 92.46%.

### 2.4 Ethical approval

This study was approved by the Medical Ethics Committee of the Affiliated Hospital of Zunyi Medical University (Approval No.: KLL-2024-736). All methods were conducted in accordance with the ethical principles outlined in the Declaration of Helsinki and relevant national research regulations in China.

### 2.5 Statistical analysis

Mplus 8.3 software was used for LPA. The scores of the 32 items from the excessive oxygen therapy scale were used as observed indicators to establish LPA models with 1–4 profiles successively. The optimal model was determined based on model fit indices. The Akaike Information Criterion (AIC), Bayesian Information Criterion (BIC), and sample-adjusted BIC (aBIC) values were used, with smaller values indicating better model fit. The entropy value ranges from 0 to 1, with values closer to one indicating higher classification accuracy, the value ≥ 0.8 indicates a model classification accuracy of 90%. The Lo-Mendell-Rubin likelihood ratio test (LMR) and the Bootstrapped likelihood ratio test (BLRT) were also used. A *P* < 0.05 indicates that the K-class model is better to the (K-1)-class model (19). SPSS 23.0 software was used for data processing. Measurement data were described using the median and interquartile range based on normality test results. Count data were described using frequencies and percentages. Chi-square tests and ANOVA were used for univariate analysis, as well as logistic regression analysis to identify influencing factors of latent profiles of nurses’ KAP regarding excessive oxygen therapy (*P* < 0.05).

## 3 Results

### 3.1 Participants’ characteristics

A total of 687 nurses were included in this study, with an average age of 31.82 ± 5.32 years. Regarding educational background, the majority of participants held a bachelor’s degree or higher, accounting for 93.74% collectively. The most common professional title was senior nurse or above, comprising 40.03% of the sample. 85.12% of the participants were female. 84.13% of the nurses worked in tertiary hospitals. Other general demographic data are presented in Table 1.

**TABLE 1:** Demographic characteristics of participants (*n* = 687).

Variable	Frequency (n)	Percentage (%)
**Age(year)**	31.82 ± 5.32^a^	
Gender		
Male	93	14.88
Female	594	85.12
Professional title		
Nurse	190	27.66
Senior nurse	222	32.31
Supervisor and above	275	40.03
Education		
Associate degree	43	6.26
Bachelor’s degree or above	644	93.74
Hospital level		
Tertiary hospital	578	84.13
Secondary hospital	109	15.87
Department		
Internal medicine	173	25.18
Surgery	92	13.92
ICU	26	3.78
Emergency department	295	42.40
Others	101	14.17
Work experience	9.11 ± 5.45^a^	
Oxygen therapy training		
Yes	258	37.55
No	429	62.45

*^a^*M ± SD.

### 3.2 Latent profiles of excessive oxygen therapy among nurses

A four model was finally fitted in this study. The model fit indices are presented in Table 2. With an increase in the number of classes, the values of AIC, BIC and aBIC decreased gradually. Critically, when moving from the three-class to the four-class model, the LMR test (*P* = 0.0902) and BLRT test (*P* = 0.0964) were both non-significant, indicating that the four-class model does not provide a statistically significant improvement over the three-class model. Therefore, following the principle of parsimony, the three-class model is preferred. This statistical evidence is further supported by the maximum entropy value of the three-class model (0.841), as well as a reasonable class probability distribution (19.65, 20.23, and 60.12%) with no excessively small classes. Moreover, the three-class solution yielded distinct and practically meaningful differences in nurses’ KAP regarding excessive oxygen therapy, facilitating a more detailed understanding of heterogeneity. The stability of the classification was also confirmed, as the average posterior membership probabilities were 0.914 for C1, 0.863 for C2, and 0.946 for C3, presented in Table 3, all exceeding the recommended threshold of 0.80. Taken together, the three-class model was selected as the optimal latent profile model.

**TABLE 2:** Comparison of fit parameter indices of different latent profile models (*n* = 687).

Model	LL	AIC	BIC	aBIC	Entropy	LMR	BLRT	Category probability (%)
C1	−5858.736	11729.472	11756.666	11737.615	–	–	–	–
C2	−5750.636	11521.272	11566.596	11534.844	0.832	< 0.001	<0.001	77.88/22.12
C3	−5690.718	11409.435	11472.888	11428.436	0.841	<0.001	0.0001	19.65/20.23/60.12
C4	−5668.654	11373.308	11454.890	11397.737	0.890	0.0902	0.0964	22.85/49.64/12.96/14.56

LL, Log-likelihood value; AIC, Akaike Information Criterion; BIC, Bayesian Information Criterion; aBIC, Adjusted Bayesian Information Criterion; Entropy, A measure of classification uncertainty; LMR, Likelihood Ratio; BLRT, Bayesian Likelihood Ratio Test.

**TABLE 3:** Average membership probabilities for each latent profiles of participants (*n* = 687).

Potential profiles	C1	C2	C3
C1	0.914	0.065	0.021
C2	0.137	0.863	0.000
C3	0.054	0.000	0.946

**FIGURE 1:**
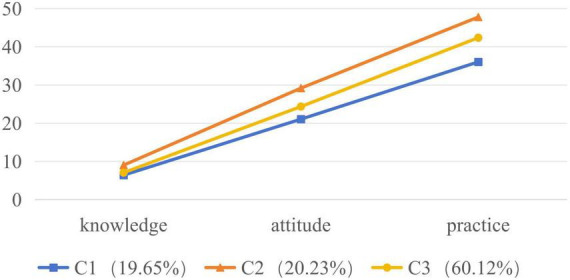
Subgroup proportions of latent profiles analysis for excessive oxygen therapy among nurses.

Based on the fitting of the latent profile model, three distinct profiles of nurses’ KAP regarding excessive oxygen therapy were identified across the three dimensions, as illustrated in Figure 1. Among them, C3 had the largest proportion (60.12%), with a score of 7.046 in the knowledge dimension, 24.346 in the attitude dimension and 42.365 in the behavior dimension. All scores fell at the intermediate level among the three groups, reflecting a moderate level of knowledge, a moderately positive attitude and a moderately level of practice, thus, this profile was named the “Moderate Knowledge-Moderately Attitude-Moderate Practice” group. C1 accounted for 19.65%, with the lowest knowledge score (6.400) among the three groups, a moderately attitude score (21.046), and a similarly low practice score (36.027), therefore, C1 was named the “Low Knowledge-Moderately Attitude-Moderate Practice” group. C2 accounted for 20.23%, with the highest knowledge score (9.013), attitude score (29.167) and practice score (47.764), therefore, C2 was named the “High Knowledge-Positive Attitude-High Practice” group.

### 3.3 Univariate analysis of factors influencing the latent profiles of nurses’ kap regarding excessive oxygen therapy

The three latent profiles of nurses’ KAP regarding excessive oxygen therapy exhibited statistically significant differences in terms of age, professional title, educational background, hospital level, department, work experience, and oxygen therapy training (*P* < 0.05), as presented in Table 4.

**TABLE 4:** Univariate analysis of latent profiles (*N* = 687).

Variable	C1(*n* = 135)	C2(*n* = 139)	C3(*n* = 413)	χ^2^/*F*	*P*
**Age(year)**	28.36 ± 5.32	36.15 ± 3.48	31.50 ± 4.85	150.629	< 0.001
**Gender**				1.302	0.522
Male	15(2.2%)	22(3.2%)	56(8.2%)		
Female	120(17.5%)	117(17.0%)	357(52.0%)		
**Professional title**				286.666	< 0.001
Nurse	97(14.1%)	2(0.3%)	91(13.2%)		
Senior Nurse	34(4.9%)	17(2.5%)	171(24.9%)		
Supervisor and above	4(0.6%)	120(17.5%)	151(22.0%)		
**Education**				80.071	< 0.001
Associate degree	31(4.5%)	2(0.3%)	10(1.5%)		
Bachelor’s degree or above	104(15.1%)	137(19.9%)	403(58.7%)		
**Hospital level**				101.689	< 0.001
Tertiary hospital	76(11.1%)	134(19.5%)	368(53.6%)		
Secondary hospital	59(8.6%)	5(0.7%)	45(6.6%)		
**Department**				77.432	< 0.001
Internal Medicine	54(7.9%)	27(3.9%)	92(13.4%)		
Surgery	38(5.5%)	15(2.2%)	39(5.7%)		
ICU	4(0.6%)	9(1.3%)	13(1.9%)		
Emergency Department	23(3.3%)	60(8.7%)	212(30.9%)		
Others	16(2.3%)	28(4.1%)	57(8.3%)		
**Years of work experience**	5.69 ± 5.28	13.79 ± 3.70	8.65 ± 4.95	16.283	< 0.001
**Oxygen therapy training**				58.422	< 0.001
Yes	71(10.3%)	15(2.2%)	172(25.0%)		
No	64(9.3%)	124(18.0%)	241(35.1%)		

### 3.4 Multivariate analysis of factors influencing the latent profiles of nurses’ KAP regarding excessive oxygen therapy

Taking the three latent profiles of nurses’ KAP regarding excessive oxygen therapy as the dependent variable (with C1 as the reference), we designated the statistically significant factors screened by univariate analysis as independent variables for a binary logistic regression analysis. The results showed that in the comparison between C1 and C2: nurses with professional titles at or below senior nurse, associate degree, or no experience of oxygen therapy training were more likely to be classified into C1 (*P* < 0.05), and nurses with older age, longer work experience, or employment in tertiary hospitals were more likely to be assigned to C2 (*P* < 0.05). In the comparison between C1 and C3: nurses with professional titles at or below senior nurse, associate degree, or no experience of oxygen therapy training were more likely to classified into C1 (*P* < 0.05), and nurses with older age, longer work experience, or employment in tertiary hospitals or working in the ICU were more likely to be categorized into C3 (*P* < 0.05). Details are presented in Table 5.

**TABLE 5:** Logistic regression analysis of factors influencing excessive oxygen therapy among nurses (*N* = 687).

Variable	β	SE	Wald χ^2^	*P*	*OR*	95%CI	
						Lower	Upper
C1 vs. C2							
**Intercept**	−9.691	1.538	39.719	< 0.001			
**Age(year)**	2.152	0.411	27.408	< 0.001	8.600	3.843	19.247
**Years of work experience**	1.691	0.379	19.910	< 0.001	5.427	2.582	11.407
Professional title							
Nurse	−3.582	1.067	11.266	0.001	0.028	0.003	0.225
Senior nurse	−2.674	0.745	12.875	< 0.001	0.069	0.016	0.297
Supervisor and above (reference)							
Education							
Associate degree	−4.844	1.152	17.670	< 0.001	0.008	0.001	0.075
Bachelor’s degree or above (reference)							
Hospital level							
Tertiary hospital	5.426	0.721	56.619	< 0.001	227.176	55.282	933.556
Secondary hospital (reference)							
Department							
Internal Medicine	−0.225	0.691	0.106	0.745	0.799	0.206	3.097
Surgery	−0.520	0.814	0.408	0.523	0.595	0.121	2.933
ICU	−1.264	1.135	1.239	0.266	0.283	0.031	2.615
Emergency department	0.885	0.672	1.734	0.188	2.424	0.649	9.056
Others(reference)							
Oxygen therapy training							
Yes	−4.181	0.536	60.874	< 0.001	0.015	0.005	0.044
No(reference)							
C1 vs. C3							
**Intercept**	−3.092	1.214	6.486	0.011			
**Age (year)**	1.355	0.337	16.133	< 0.001	3.878	2.002	7.512
**Years of work experience**	0.900	0.313	8.243	0.004	2.459	1.330	4.544
Professional title							
Nurse	−1.480	0.689	4.615	0.032	0.228	0.059	0.878
Senior nurse	−1.227	0.668	3.377	0.066	0.293	0.079	1.085
Supervisor and above (reference)							
Education							
Associate degree	−4.956	0.757	42.856	< 0.001	0.007	0.002	0.031
Bachelor’s degree or above(reference)							
Hospital level							
Tertiary hospital	3.444	0.479	51.785	< 0.001	31.306	12.254	79.978
Secondary hospital (reference)							
Department							
Internal Medicine	−0.81	0.544	1.142	0.285	0.559	0.193	1.624
Surgery	−1.020	0.633	2.595	0.107	0.361	0.104	1.247
ICU	−1.931	0.962	4.030	0.045	0.145	0.022	0.955
Emergency department	1.027	0.573	3.212	0.073	2.791	0.908	8.578
Others (reference)							
Oxygen therapy training							
Yes	−1.944	0.409	22.533	< 0.001	0.143	0.064	0.319
No(reference)							

## 4 Discussion

LPA was used to examine the heterogeneity in nurses’ KAP regarding excessive oxygen therapy. The results identified three distinct KAP profiles. To the best of our knowledge, this is the first study to apply LPA to investigate the heterogeneity of nurses’ KAP regarding excessive oxygen therapy. The findings suggest that demographic factors, including age, education, work experience, professional title, hospital level, department, and oxygen therapy training, may exert a significant influence on nurses’ KAP levels related to excessive oxygen therapy.

### 4.1 Heterogeneity exists in nurses’ KAP regarding excessive oxygen therapy

Previous studies (6, 13, 22) have demonstrated a consistent disparity between knowledge, attitude, and practice (KAP) among nurses regarding oxygen therapy, indicating that positive attitudes and proactive practice do not always equate with sufficient knowledge. In addition, marked variations in KAP levels have been reported across different regions, which is in line with the findings of the present study. Our results further identified distinct heterogeneity in nurses’ KAP related to excessive oxygen therapy, characterized by three unique profiles: “Low Knowledge–Moderate Attitude–Moderate Practice,” “Moderate Knowledge–Moderate Attitude–Moderate Practice,” and “High Knowledge–Positive Attitude – High Practice.” These distinct subgroups reflect clinically relevant differences in nurses’ KAP regarding excessive oxygen therapy, which are directly linked to oxygen safety, maintenance of optimal patient oxygenation, and the risk of adverse clinical events. Existing evidence confirms that adequate knowledge of oxygen therapy is strongly associated with the quality of critical care nursing, and adherence to evidence-based best practices and standardized protocols is essential to ensure therapeutic efficacy and improve patient outcomes (16). Accordingly, we recommend that nursing educators provide regular and targeted training on oxygen therapy, including systematic interpretation of international and domestic guidelines and expert consensuses, to disseminate up-to-date concepts and evidence. Such initiatives will promote rational oxygen administration, enhance the quality of oxygen therapy, and ultimately safeguard patient safety during oxygen delivery (18).

### 4.2 Factors influencing the heterogeneity in nurses’ KAP regarding excessive oxygen therapy

#### 4.2.1 Age and work experience

The results of this study show that as age and years of work experience increase, nurses were more likely to classified into the “High Knowledge-Positive Attitude-High Practice” group. Some studies had indicated that healthcare workers with more than 4 years of experience have better knowledge of oxygen therapy (6), while others had found that nurses with ≥ 10 years of experience possess better knowledge (13). Our study did not categorize years of work experience, which resulted in the failure to identify the specific impact of different stages of work experience on nurses’ KAP regarding excessive oxygen therapy. Future longitudinal studies could be conducted to explore the influence of work experience on this construct. However, several studies have failed to find a significant association between nurses’ work experience and their mastery of oxygen therapy knowledge (23, 24). Kimario et al. reported findings inconsistent with those of the present study, demonstrating no significant correlation between age and nurses’ practice related to oxygen therapy (10, 23). These inconsistent results suggest that clinical competence should not be improved by work experience and age alone, multiple factors such as job satisfaction, work stress, and critical thinking must also be considered (25).

#### 4.2.2 Professional title

In our study, nurses with higher professional titles were more likely to be classified into the “Moderate Knowledge-Moderately Attitude-Moderate Practice” group, with a probability over four times greater than belonging to the “High Knowledge-Positive Attitude-High Practice” group. These findings indicated that a higher professional title does not necessarily correlate with better levels of KAP regarding excessive oxygen therapy among nurses. This may be explained by the relationship between professional title and job responsibility, for instance, senior professional titles often involve additional administrative duties, which can reduce hands-on clinical practice and frontline work. However, Ke et al. (26) reported that higher professional titles were linked to a lower incidence of excessive oxygen therapy.

In contrast, Zhao et al. (13) found no significant association between professional title and KAP related to oxygen therapy. These findings suggest that additional influencing factors beyond professional title should be taken into account when designing and implementing oxygen therapy-related training for nurses.

#### 4.2.3 Education level

Our findings revealed that nurses with an associate degree had a significantly higher risk of excessive oxygen therapy compared to those with a bachelor’s degree or higher (C1 vs.C2: OR = 0.008, *P* < 0.001; C1 vs. C3: OR = 0.007, *P* < 0.001), which demonstrates the decisive role of educational level in nurses’ risk of excessive oxygen therapy. This finding is consistent with the study by Ke et al. (26). This may be attributed to the fact that undergraduate education places greater emphasis on theoretical knowledge, the cultivation of evidence-based thinking and the construction of a systematic knowledge framework, whereas college education focuses more on practical skill training (27), Such educational disparities may result in relatively insufficient knowledge reserves among nurses with a college diploma, thereby increasing their risk of excessive oxygen therapy. However, Zhao et al. (13) found that nurses with a college diploma possessed better knowledge of excessive oxygen therapy than those with a bachelor’s degree or above, which may be related to differences in research design, study population, evaluation indicators, and measurement tools. Overall, higher-level academic education helps improve nurses’ mastery of oxygen therapy indications and risk control, thereby reducing non-standard oxygen therapy behaviors. Therefore, clinical training and continuing education should be strengthened for nurses with lower educational qualifications to narrow the practice gap caused by educational differences and improve the safety of oxygen therapy.

#### 4.2.4 Oxygen therapy training

Compared with the “Low Knowledge-Moderate Attitude-Moderate Practice” group, nurses who had participated in oxygen therapy training demonstrated better KAP levels than those who had not, highlighting the importance of training. The “Low Knowledge-Moderate Attitude-Moderate Practice” group accounted for 19.65% in this study, which was lower than that reported in previous studies (7, 28), This discrepancy may be attributed to differences in sample size, research setting, and methodology. Kane et al. noted that nurses had knowledge gaps regarding oxygen therapy, emphasizing the need to master core oxygen therapy-related knowledge, including its indications, normal oxygen saturation across different age groups, and normal respiratory rates (29), Accordingly, it is recommended that nursing managers and educators strengthen oxygen therapy knowledge training for nurses.

#### 4.2.5 Hospital level

The results indicated a significant association between hospital level and nurses’ KAP related to excessive oxygen therapy, with nurses in tertiary hospitals showing significantly higher KAP levels (OR = 227.176, *P* < 0.05). On the one hand, secondary hospitals are limited by shortages in human resources, educational resources, and medical equipment, which directly hinder nurses from acquiring and mastering new knowledge. On the other hand, tertiary hospitals have a larger base of patients receiving oxygen therapy, and nurses in these settings can more frequently observe the immediate adverse consequences of nonstandard oxygen therapy, thus accumulating more extensive experience in oxygen therapy management in clinical practice. Notably, the relatively large odds ratio may be influenced by sparse data distribution and unbalanced composition between groups, suggesting potential sparse data bias. Therefore, the interpretation of its effect size should be more cautious, with emphasis placed on the statistical association trend. Therefore, it is essential to strengthen training on excessive oxygen therapy for nurses in primary hospitals. With the backdrop of policies such as the medical alliance, tertiary hospitals should fully play a leading role and provide regular training on the latest medical knowledge to primary hospitals. For instance, experts can be selected and dispatched to primary hospitals to conduct teaching and guidance (18).

#### 4.2.6 Department

The study by Ke et al. (21) demonstrated that 56.19% of ICU nurses had a moderate knowledge score, 72.81% held a neutral attitude, and 59.69% exhibited moderate practice, which was consistent with the findings of our study. ICU nurses were more likely to be classified into the “Moderate Knowledge-Moderately Attitude-Moderate Practice” group (OR = 0.145, *P* = 0.045). This may be attributed to the fact that ICU patients are critically ill and that ICU nurses frequently administer oxygen therapy and have received relevant training such as updates on oxygen therapy guidelines and high-flow oxygen therapy (30).

### 4.3 Limitations

This study has several limitations. First, as a cross-sectional survey conducted via the Wenjuanxing platform, the findings cannot establish a causal relationship between the influencing factors and nurses’ KAP regarding excessive oxygen therapy. Second, the study participants were restricted to one province in China, which may limit the generalizability of the results. Subsequent multi-center investigations can be conducted to address this limitation. Third, in this study, the odds ratio between hospital level and KAP level was large (OR = 227.176), which may indicate the presence of sparse data bias, leading to unstable effect size estimation. This finding requires further validation in larger samples. Furthermore, owing to the constraints of the study design and data collection, we were unable to account for all potential factors associated with nurses’ KAP related to excessive oxygen therapy. Therefore, future studies should incorporate additional variables, such as organizational support and work environment, to further explore this topic.

## 5 Conclusion

This study confirms that nurses’ KAP regarding excessive oxygen therapy can be categorized into three profiles, and demographic-related characteristics significantly influence their KAP. The findings provide an important theoretical basis for nursing managers and educators to develop relevant training programs.

## Data Availability

The raw data supporting the conclusions of this article will be made available by the authors, without undue reservation.
